# Correction: “Acute kidney injury in critically ill patients with COVID–19: The AKICOV multicenter study in Catalonia”

**DOI:** 10.1371/journal.pone.0295778

**Published:** 2023-12-07

**Authors:** Arsenio De La Vega Sánchez, Ana Navas Pérez, Marcos Pérez-Carrasco, María Torrens Sonet, Yolanda Diaz Buendia, Patricia Ortiz Ballujera, Miguel Rodríguez López, Joan Sabater Riera, Aitor Olmo-Isasmendi, Ester Vendrell Torra, María Álvarez García-Pumarino, Mercedes Ibarz Villamayor, Rosa María Catalán Ibars, Iban Oliva Zelaya, Javier Pardos Chica, Conxita Rovira Anglès, Teresa M. Tomasa-Irriguible, Anna Baró Serra, Edward J. Casanova, Francisco J. González De Molina, on behalf of The AKICOV Group

The 15^th^ author’s name is spelled incorrectly. The correct name is: Javier Prados Chica.
