# Supplementary material for: “Acute kidney injury in critically ill patients with COVID–19: The AKICOV multicenter study in Catalonia”
Source: PLoS One. 2023 Apr 14;18(4):e0284248. doi: 10.1371/journal.pone.0284248 (PMC10104297; doi:10.1371/journal.pone.0284248)
Supplement: Attachment — Submitted filename: Response to Reviewers.docx [file pone.0284248.s001.docx]

**Response to Reviewers**

Dear Reviewers of PLOS One,

We would like to express our gratitude for taking the time to review our article titled "Acute kidney injury in critically ill patients with COVID-19: the AKICOV multicenter study in Catalonia." Your constructive feedback and suggestions were extremely helpful in improving the quality of the article.

We are writing to inform you of the changes we made based on your feedback:

1- “Avoid abbreviations not explained in the abstract” and “Also in the text when using the abbreviations explain them the first time they are used, see RRT”. We have explained all abbreviations used in the abstract and manuscript, as suggested in your comments.
2- “The material and method need to be defined: ARDS, organic dysfunction”. We included definitions for all the dysfunctions used in the study, as you recommended.
3- “Describing the usual treatments of the patients and those used during admission brings originality to the study, there are few that describe them”. We have expanded the discussion section to include a more detailed description of the usual treatments given to patients and those used during admission, as you noted that few studies have done so.
4- “Have differences been observed between the participating hospitals in terms of these treatments?”. We have explained in the discussion that no significant differences were detected between ICUs, likely due to a regional unified protocol being established.

Yours faithfully,

Marcos Pérez Carrasco	Arsenio de la Vega Sánchez
